# “I’m 'just' a community pediatrician” views and challenges of pediatricians working in the community in Israel

**DOI:** 10.1186/s13584-023-00563-y

**Published:** 2023-04-17

**Authors:** Shulamit Pinchover, Rony Berger-Raanan, Maya Yaari, Mary Rudolf, Lisa Rubin, Dafna Idan-Prusak, Vera Skvirsky, Tirzah Margolin, Hava Gadassi

**Affiliations:** 1 Ghoshen, Haruv Campus, Mt Scopus, Jerusalem, Israel; 2 Azrieli Faculty of Medicine, Bar Ilan University, Ramat Gan, Israel; 3 School of Public Health, University of Haifa, Haifa, Israel; 4 Rashi Foundation, Jerusalem, Israel

**Keywords:** Community, Pediatrics, Professional identity, Challenges, Stature, Isolation

## Abstract

**Background:**

There are ongoing changes around the world in the training and practice of pediatricians who work in the community. These changes are driven by the understanding that pediatricians are required to provide not only acute primary care but also to address more comprehensive concerns, particularly the ‘new morbidities’. The present study examines the professional identity of Israeli pediatricians in the community in light of these changes, the barriers and challenges to their work and professional adaptations in the field.

**Methods:**

We used a mixed-methods approach, collecting the perspectives of 137 pediatricians who work in the community through an anonymous online survey, followed by in-depth semi-structured interviews with 11 community pediatricians.

**Results:**

The survey results show that community pediatricians in Israel have limited knowledge on a variety of developmental, behavioral and emotional issues; that they lack working relationships with medical or other professionals; and are rarely engaged with other community services. Three main themes arose from the interviews that support and deepen the survey results: perceptions of the profession (pediatrics in the community vs. community pediatrics), the stature of pediatricians in the community (during residency, the choice to work in the community, their daily work) and barriers and change in community pediatrics (isolation, limited resources and challenges arising from the nature of community work).

**Conclusions:**

The present study sheds light on the professional identity and the day-to-day challenges and successes of pediatricians working in the community. Continuing medical education, providing a supportive framework and professional community, better resources, more time with patients, and tools and opportunities for professional development would help pediatricians who work in the community to overcome some of these challenges. The research findings reinforce the need for policy change in the field of community pediatrics with a specific community training curriculum, provision of more resources and ongoing support for pediatricians. This requires partnership between the HMOs, the Ministry of Health, the Scientific Council (Israel Medical Association, professional organizations) and NGOs in order to turn individual-level solutions into system-level and policy-changing solutions.

## Background

There is an increasing emphasis on the need to implement the bio-psycho-social model in the field of pediatrics, and particularly in community pediatrics [1, 2]. While children’s acute morbidity rates have been declining in recent years [3], pediatricians who work in the community are required to provide not only traditional acute primary care but also to address more comprehensive health issues such as the ‘new morbidities’[4]. These include emotional, behavioral and developmental problems, parenting difficulties, child maltreatment, chronic childhood illnesses, obesity, sexuality and identity, engagement in community health promotion and more [5, 6]. These morbidities may be considered low in severity, but are very prevalent and can impact significantly upon overall developmental outcomes and health later in life. In Israel, as in many other countries, the nature of pediatric training is still predominantly based in hospital, with a focus on physical illness [7]. The appropriate tools, skills and exposure required for community pediatric work [5, 8] are inadequately provided. An Israeli study regarding the management of psychosocial issues found that many pediatricians who work in the community face psychosocial issues in their practice, without having the training, time or confidence to provide the support and treatment needed [9].

Around the world there are ongoing changes in the training and practice of doctors providing community primary care for children [10–12]. The American Academy of Pediatrics (AAP) definition of community pediatrics expands the focus from one child to all children in the community. It recognizes that family, educational, social, cultural, spiritual, economic, environmental, and political forces have significant effects on the health and of children. It synthesizes clinical practice and public health principles directed toward providing and promoting health of all children in all contexts; and has the commitment to use the local community’s resources in collaboration with other professional community members [13]. These principles can be translated into three aspects in the pediatricians’ work: (1) clinical work that includes all aspects of the new morbidities, as described above, (2) collaborative interprofessional work and (3) community activism and involvement. In order for pediatricians to act on these aspects, change in professional identity and structure needs to be made.

Professional identity comprises various components, structural and personal. In Israel, as in many other Western countries, there is a shortage of pediatricians in the community [14]. Beyond the obvious reasons of population growth and shortage of training positions, there is difficulty in attracting pediatricians to community work [15]. For the most part, there is a hierarchical ranking of professional prestige in the medical world between specializations and fields of medicine. Community medicine is often perceived as less prestigious, less challenging and less innovative than hospital medicine^1^ [16]. In less prestigious fields of medicine, physicians face lower reputation, higher workload, feelings of professional isolation, limited career development opportunities and high burnout [17, 18]. These, in turn, may affect professional identity and make doctors less satisfied and less likely to stay in their position, which leads to health service workforce imbalances and suboptimal health outcomes, especially in primary care areas such as pediatrics [16]. It can also make it harder to implement new models for community pediatrics and expand pediatrician responsibilities.

Beyond the professional framework, professional identity develops within the daily work, its barriers and its challenges. The Social and Cognitive Career Theory (SCCT) [19, 20] emphasizes the barriers in professional development which may interfere with professional tendencies and achievement of professional goals, but which may also be a motivator for change and development [21, 22]. For pediatricians who work in the community, these barriers may be related to changes in the profession, characteristics of the community, the work environment, or the physicians themselves [9]. In light of the unmet needs of children facing the ‘new morbidities’ and the ongoing changes in pediatrics in the community in Israel, the present study examines the professional identify of pediatricians in the community, in relation to the current definitions of community pediatrics, the barriers and challenges of their work and the ways in which they create changes in the field. The study was conducted by Goshen, a non-profit organization established in 2014 that focuses on changing the nature of pediatric healthcare services in the community in Israel through training, mentoring and policy change [23].

## Methods

We used a mixed-methods approach, collecting the perspectives of pediatricians who work in the community through an anonymous online exploratory survey, that informed the structure of in-depth semi-structured interviews with a smaller sample of pediatricians. The study was approved by the ethics committee of the Department of Psychology at the University of Haifa.

### Exploratory survey

An exploratory survey aims to provide insights on a previously unexplored topic and which does not pursue statistical representativeness of the results. The current survey aimed to gain preliminary knowledge and understanding into practical aspects related to the work of pediatricians in the community.

#### Sample

We conducted a non-representative survey with pediatricians who work in the community.

#### Measures

The survey included 4 questions regarding: (a) Knowledge and skills about issues in community pediatrics [“*To what extent do you have the knowledge and tools needed to address the following issues*”, on a scale of 1–5 (1 = not at all, 5 = extremely knowledgeable)]. (b) The relationship with other professionals in the community [“*During the past year, did you consult with the following professionals?*”, (0 = no 1 = yes)]. (c) Involvement in the community [“*During the past year, were you involved in the following activities in the community?”* (0 = no 1 = yes)]. (d) Barriers in practicing community pediatrics *(“Of the following, what are the three major barriers in implementation of community pediatrics practice in Israel?”*).

#### Procedure

The survey was distributed on professional networks (Goshen’s email list^2^) and social media (Goshen Telegram group^3^) during 2021. The email list includes 1733 pediatricians who work in the community, out of 2028 doctors who worked as pediatricians in the community at that time. It is not possible to calculate the response rate as it is not known how many contacts were out of date or duplicated. However it was likely to be in the range of 10%, the range typically anticipated in online exploratory surveys [24]. Data was collected anonymously using eSurvey. Descriptive statistics were used to analyze the survey data with SPSS 23.0.

### Interviews

#### Sample

A request to participate in the interviews was disseminated through Goshen’s email list, during 2021. From those who expressed interest (42), participants were selected based on gender, sectoral affiliation and seniority, in order to produce an appropriately diverse sample. Eleven pediatricians who work in the community were interviewed. Mean age was 51.5 years, (*SD* = 9.2), and years in practice in community work was 15.4 (*SD* = 10.9) (see Table 1 for full characteristics).

**Table 1:** Demographic characteristic of interviewees

Name	Age	Gender (F = female, M = male)	Sector (A = Arab, J = Jewish)	Years in Practice
C	68	F	J	33
A	62	F	J	30
H	60	M	A	30
Sh	56	F	J	8
Y	52	F	J	15
T	51	F	J	16
M	47	M	J	7
R	47	F	J	12
S	44	M	J	10
J	42	F	J	7
N	38	F	A	1

#### Measures

The interview guide included open-ended questions on the work environment, work routine and barriers and challenges in the community work and community initiatives.

#### Procedure

The interviews were conducted by three authors (RB-R, HG and DI-P). At the beginning of each interview, the interviewers explained about Goshen and the research’s purpose. All participants signed an informed consent form. The interviews were conducted by phone or zoom, and each interview lasted between 60 and 90 min, was recorded and transcribed. All names were changed to maintain confidentiality and anonymity. Interviews were analyzed using thematic qualitative analysis. The researchers performed a theory-oriented content analysis, and created a list of codes into which the interviewees' words were sorted and from which the main themes in the study were developed. The credibility of the study was verified by double analysis of seven of the interviews by two researchers independently and data was collected to the point it reached saturation.

## Results

### Survey results

137 pediatricians who work in the community participated in the survey. Years in practice in pediatrics ranged between 1 and 45 years (*m* = 14.9, *SD* 11.3).

#### Clinical knowledge/clinical confidence

**Fig. 1:**
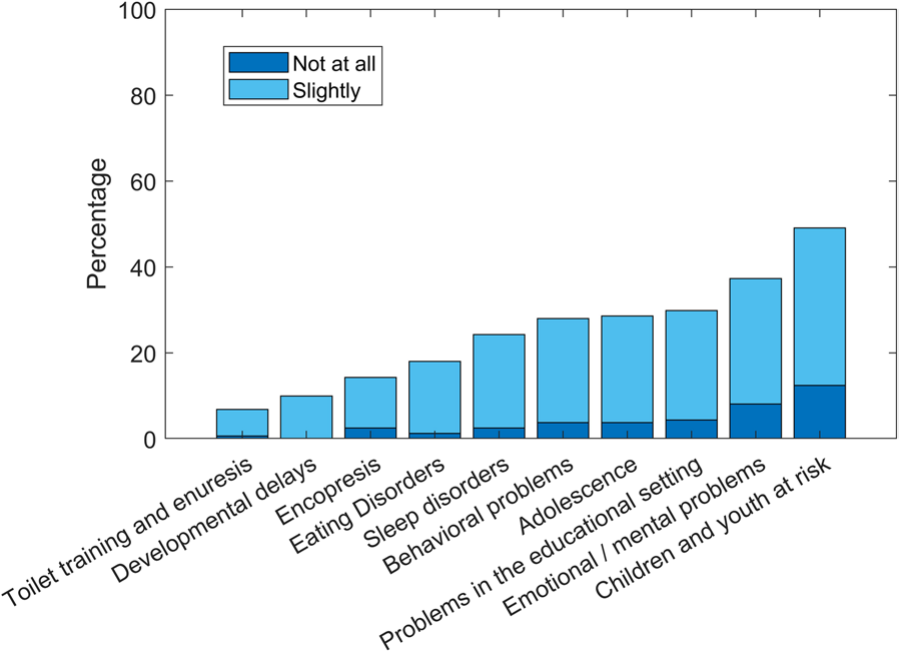
Percentages of pediatricians’ “not at all” or “slightly” responses to the question “*To what extent do you have the knowledge and tools needed to address the following issues*”

Given the prevalence of psychosocial concerns [6], we asked the participants how knowledgeable and skillful they felt regarding common psychosocial issues (see Fig. 1). Areas where participants reported they felt relatively knowledgeable and skillful were toilet training and enuresis (*M* = 3.53, *SD* = 0.77), developmental delays (*M* = 3.43, *SD* = 0.77) and encopresis (*M* = 3.27, *SD* = 0.86). Participants felt somewhat less confident regarding eating and sleeping problems (*M* = 3.11, *SD* = 0.82. *M* = 3.00, *SD* = 0.88, respectively). Problems in educational settings (M = 2.87 SD = 0.90), behavioral problems (M = 2.84, SD = 0.83), adolescents’ health (M = 2.83, SD = 0.84), emotional and mental health problems (M = 2.62, SD = 0.88) and children and youth at risk (M = 2.37, SD = 0.87) were the areas where participants felt least confident.

#### Interprofessional consultations

**Fig. 2:**
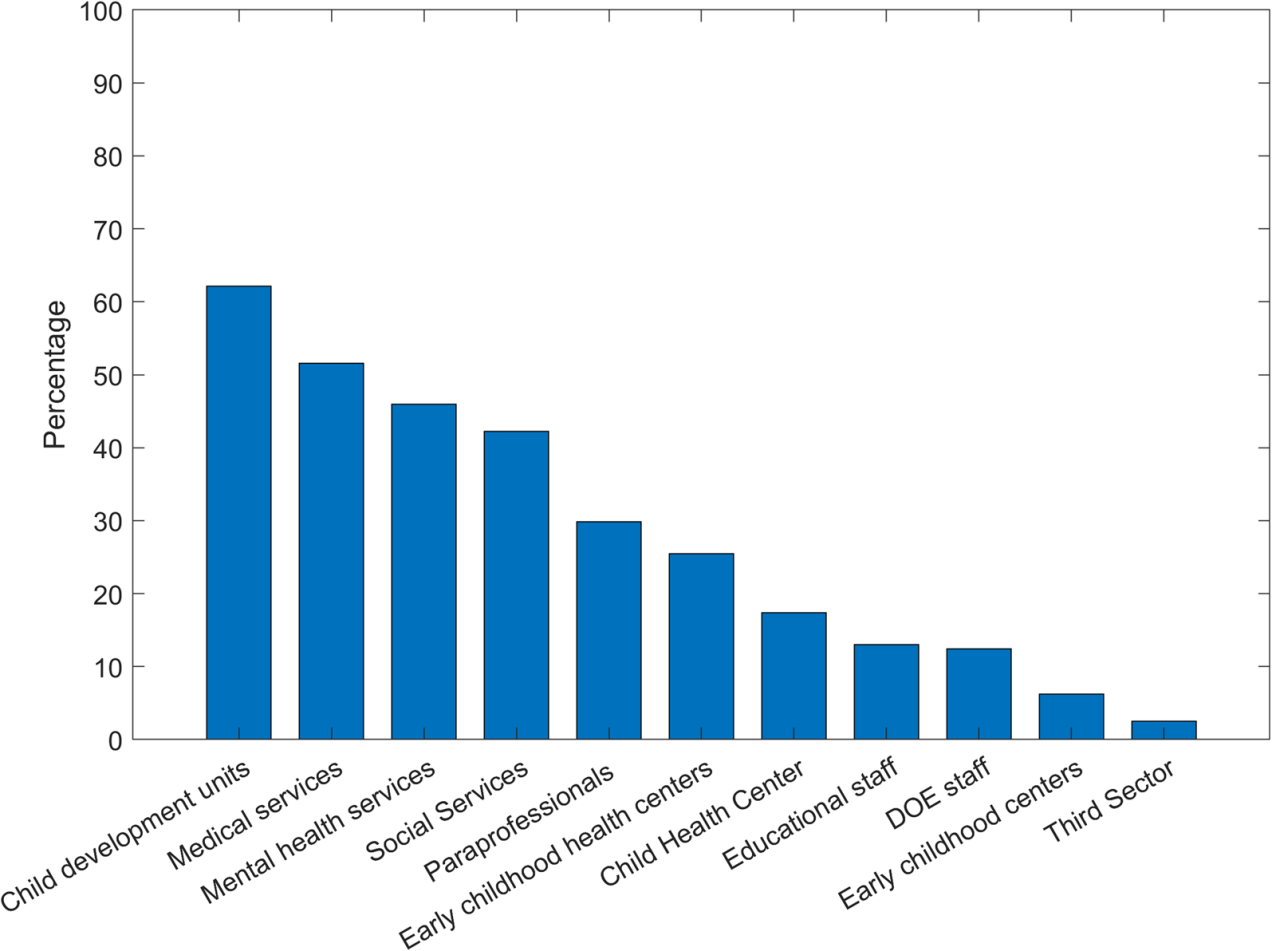
Percentages of pediatricians who, during the past year, consulted with the following professionals in their area regarding psychosocial issues

When asked about interprofessional relationships (Fig. 2), 62% reported they had consulted with child development centers in the past year, 52% had consulted with other medical professionals, 46% with social services and 42.5% with mental health professionals. One third of the participants consulted with allied health professionals (such as speech, occupational or physio therapy) and only one quarter (25%) reached out to maternal-infant health centers (Tipat Halav). Lastly, 17.5% consulted child health centers (Merkaz Bri’ut Hayeled), and even fewer consulted educational staff (13%), Department of Education (DOE) staff (12.5%), early childhood centers (6%) and professionals from the voluntary sector (2.5%).

#### Community activism and involvement

Participants were asked about their involvement in the community. Only 21% reported that they provided advice to other professionals, and 16% said they gave lectures for parents. About 8% had given a lecture in an educational or in informal settings. Only 5% participated in a multidisciplinary professional local committee and 3% were involved in a professional online forum.

When asked about the major barriers to community involvement, lack of time was offered as the main reason (with 46% responding that they lack sufficient time in the clinic, and 41% lack time to offer other services in the community). Lack of training and knowledge was also reported as a significant barrier (40%). Lack of coordination with community services and lack of familiarity with these services were identified as a major barrier by 46% and 25% of the participants respectively, 18% identified lack of remuneration for community work as a barrier, and 13% identified lack of support from the HMOs as a barrier. Seven percent of the participants reported that the way they are regarded by other professionals affects their involvement, 3% reported that the fact that it does not affect their reputation is a barrier to involvement in the community, and 1% felt that the way they are regarded by parents affects their involvement.

### Qualitative results from in-depth interviews

The following main themes emerged on analysis: perception of the profession; stature of the pediatrician in the community; and barriers and change in community pediatrics.

#### Perception of the profession

Participants were asked to define how they perceive their professional role. The respondents’ views regarding how engaged they were in clinical, behavioral, social and community aspects of care varied, and were analyzed into three sub-themes.**i. Pediatrics in the community**. Two of the 11 participants perceived their work as locale dependent; that is, the practice of clinical pediatric medicine in a community located clinic rather than in a hospital. As such, they defined “pediatrics in the community” as dealing with primary care including long-term medical care, but does not involve children’s development, behavior or environment. As M. said: "*I don’t look at the community as a community, I’m focused on the individual patient… patients with medical problems can contact (me), get a good solution and move on".ii. Behavioral-developmental pediatrics in the community*. About half of the participants (5) perceived their role as providing a broad, ecological response to the health and development of children and their families, while engaging in medical, developmental and psychosocial issues: *"You do not take care of the child, you take care of the family… It is a holistic medicine… it is not just fever or a runny nose” (A.).* This type of medicine is also reflected in the creation of collaborations with other professionals within the clinic, such as specialists and allied health professionals. However, these participants did not see their role in the community space outside the clinic.**iii. Community pediatrics**. Four physicians described pediatrics in the community as a profession that operates beyond the single patient and their needs, and extends into the community outside the clinic through health promotion and interprofessional collaborations. In order to fulfill this role, one must be involved in parent education, create relationships with the education and welfare systems and take part in policy change. According to H., the community pediatrician *"is the most important factor for disease prevention, health promotion, treatment, for discovering environmental elements, even ones that are unrelated to diseases".*

#### Stature of the pediatrician in the community

Participants raised concerns about the professional prestige of pediatrics in the community that is reflected in different ways in all stages of professional development, as described in the sub-themes.

**i. Residency**. According to four participants, exposure to community pediatrics during residency training is limited and unrepresentative and many times can be perceived as a negative experience. This creates an unfavorable attitude towards the profession:

"We called it (the community pediatrics rotation) the 'hole- plugging unit' ... moving you…to anywhere the system needs you at that moment ... it's a kind of trauma…(the system) basically tells you that community pediatrics is not something you want to deal with" (R.)

**ii. Choosing to work in the community**. After (or during) the residency, residents are required to choose between working in the hospital or in the community. The’exit’ from the hospital to the community is accompanied, according to four participants, by disparagement from those in the hospital, who see the work in the community as less prestigious compared to the hospital:

"The biggest challenge in going out into the community, was dealing with the ego ... how do I explain to my peers ... to the professors who mentored me and expected more from me ... that in the end I’m ‘just’ a community pediatrician ... I’m now more than 10 years in the community ... and despite the seniority and status and appreciation ... there is still some need to be apologetic ... "(Q.)

**iii. The daily work in the community**. Regarding daily work in the community, six participants experienced condescension on the part of hospital staff, which impairs professional cooperation and the provision of optimal care for children who move between community and hospital care. This in turn exacerbates the low professional prestige of pediatrics in the community:

"Inside the hospital there was a poor attitude ... even disrespect for community pediatricians… Many doctors would not even read the referral letters or would say all sorts of things about the referring pediatrician ‘Well, they don’t know, what do they know?" (S.)

#### Barriers and change in community pediatrics

Working in the community is characterize by unique challenges and barriers, which often drive deliberate and active changes of work routine and contribute to the development of the professional identity. Three main sub-themes, relating to isolation, limited resources, and challenges were identified.**i. Isolation.** Work in the community is more isolating than hospital work, which inherently involves team work. The pediatrician in the community often works alone, as a sole pediatrician in the clinic, and with no built-in contact with other colleagues.

Isolation was expressed in terms of loneliness and lack of teamwork:

“I would like ... to turn my clinic into something…that is a little more like a hospital, in which you can consult... (here) there is no joint team working together on the same patient even if it involves more complex care" (M.)

Participants also expressed a sense of professional isolation, leading to a lack of up-to-date medical knowledge, tools and skills that are needed in the community. As Y. said *“In a hospital it's constant … you study all the time. In the community you do not".*

Finally**,** 10 participants said they feel isolated and disconnected from the community they work in, and have no or minimal relationships with other professionals in the community, such as educators, welfare and mental health services or community leaders: “*I do not really have connection to the community…” (T.).*

In face of these challenges, participants described changes they made in order to feel more connected socially and professionally. Most of the changes described were individual, voluntary and piecemeal solutions rather than systemic changes, and were therefore unsustainable over time. Creating personal connections within and outside the clinic, in the community and in the hospitals, were the most common solutions. Moving to a bigger clinic, designating time to study or enrolling in an advanced course were other solutions.

"(I created) some kind of supportive social framework of people who also do a lot of preventative medicine and advocacy and education, and they are also kind of friends. But it's very specific, like it's a specific social worker and a certain dietitian."(Sh.)

**ii. Limited resources.** Limited resources of time, allied health professional services from the health maintenance organization (HMO) and burnout were perceived as major barriers.

All participants described the lack of time as a major challenge in community work, stemming from the way the Israeli health system works and remunerates physicians. According to the participants lack of time harms the quality of care, and does not allow the physician to be involved in the community. M. described that:

"At first, I had 10 minutes per patient and then I saw others do 6 (minutes), so I realized the clock needs to tick faster ... you want to make a profit, I won’t deny it".

Lack of resources is also reflected in the level of services provided by the HMO and in the HMO's perception of the pediatricians. So said R. *"For example, the fact that there is no immediate physiotherapist availability, fixes the injury, the pain … you have to come and fight (the HMO) for the patients".* The participants shared feelings of the lack of support in the practice of community work, and the financial and not professional motives of the health fund administration that limit the doctor's ability to act independently: *"We need to provide a ‘service’. We do not need to do medicine… We do not have people here, families who need our help … we have clients"* (A.)

According to 10 of the 11 participants working day after day in the clinic and limited resources creates burnout, which may lead to suboptimal treatment: *"After several hours a day I start to lose a little patience …". (M.)*

Out of a desire to provide better service, the doctors found ways to deal with the limited resources. They created personal changes in the way they communicated and dedicated their time. For example, S. said:

"I had a patient ... I almost missed a serious illness with him. Luckily in the end I sent him... to the E.R... But I felt I did not have enough time to sit with him and get all the information... you learn from such cases. You improve your communication (with parents and patients).”

Some of the participants worked at the expense of private time, trying to devote time for professional development as well as family or habits. Others were able to create an individual solution with the HMOs to get more time with each patient or more resources, but again, the solutions where piecemeal:

"'I cannot ‘box it' ... There are a lot of things I feel I'm doing right… and now I have to convince the decision makers that it's not just my obsession… that it should be replicated". (Sh.)

**iii. Challenges arising from the nature of community work.** The pediatricians in the study raised challenges stemming from the nature of the work in the community. Thus, five of the physicians in the study noted that placing boundaries between home and community work is one of the significant challenges in community work. As S. said: *"I cannot leave the clinic… when you go to the community you need … to always be available".*

Three pediatricians cited working with parents as a challenge. For example, N. said: *"If a child would come in alone to receive treatment it could have been easier for me. The other patient, the parent— I have a very hard time with them (laughs)".*

In addition, many of the physicians in the study work with diverse populations from the social periphery of Israel, which poses unique challenges: "*There are children, families that are difficult to reach … you try to explain to them how important it is but not always manage to… find a shared language.*" *(N.)*

The participants described that giving each patient more time, learning better communication strategies with parents, and gaining experience, they were able to overcome some of these barriers. For example, A. said *"I was very far from this world, this population…it wasn’t easy for me… Until I started… to understand and accept … and I saw a beauty of cooperation".*

## Discussion

Pediatricians play a vital role in promoting the health of children in the community and can maximize their impact by adopting and adapting the current models of community pediatrics [4, 19, 20, 25]. The present study highlights the challenges and barriers in pediatricians' work in Israel, that affect their professional identity and levels of involvement in the community. The survey results indicate that pediatricians have limited knowledge on a variety of developmental, behavioral and emotional issues. These findings complement those found in a previous study in Israel about pediatricians’ roles in management of psychosocial issues [9], and are supported by the qualitative results. Additionally, the qualitative results show that some pediatricians in the community adopt a more complex role, but the system (including training programs and HMOs) has not adapted nor supported this change. This disparity creates a sense of isolation, excessive workloads and lack of human and non-human resources which pose great challenges on the pediatricians in the community. Most of the pediatricians who participated in the survey did not have interprofessional relationships with professionals outside of the medical field and were not active in the community. Interviewees explained that they are missing the skills and resources to cooperate and work with the community in the way they would like.

However, as suggested by Social and Cognitive Career Theory (SCCT), and indicated in our interviews, it seems that these very challenges contribute to the development and shaping of the professional identity of the participants and can be perceived as facilitators for change and professional development [19, 20]. Still, the ways of dealing with the challenges were mostly personal and local, and were born from individual doctors’ initiatives. The disadvantage of these solutions is that they are person and context dependent, and cannot be generalized, scaled or preserved over time. The participants expressed the desire for more systemic change, change that would create a permanent framework for professional development, foster collaborations within the clinic and between the clinic and the community and hospitals, support physicians' community initiatives, and allocate time and resources for a more holistic and ecological practice. This would enable transforming individual solutions into sustainable changes in the system.

According to Self-determination theory (SDT) [21] work motivation moves on a spectrum ranging from amotivation (total disengagement), through extrinsic or controlled motivation (people undertake an activity under pressure, or expecting rewards to stay motivated), to intrinsic or autonomous motivation (people engage in an activity, because they find it interesting). In order to foster intrinsic motivation three psychological needs should be addressed: autonomy (the feeling of freedom to choose what one wants); competence (the feeling of being effective in whatever action one performs), and relatedness (the feeling of being connected with or belonging to and accepted by one’s community) [21]. Looking at the qualitative findings one can see that these are indeed the three things that pediatricians are struggling with in their daily work, feeling they do not have the autonomy from the HMOs to practice as they would desire, lacking confidence in the issues that are most relevant in community pediatrics and most of all do not feel relatedness, but isolation and disconnection from colleagues, other professionals and the community. This, together with the less prestigious reputation of working in the community is likely to lead to amotivation or to extrinsic motivation, and to difficulties in attracting new pediatricians to community work.

Allocation of more resources to the community, financial incentives, and designated rotation in community pediatrics have been found to be beneficial in attracting doctors toward the community, and promoting the change in field of community pediatrics [11, 26]. However, while financial incentives have a short-term positive effect on recruiting physicians for less prestigious professions and working in the community, they do not have a long-term effect on keeping doctors in the community or on changing the profession’s reputation [2]. While improving pediatric education and continuing education is necessary to fill in the gaps documented for community pediatricians [7], this study suggests that reducing the feeling of professional isolation is critical to creating an environment conducive to continuing professional development for community pediatricians. Building platforms for routine professional meetings, peer learning and consultation could reduce the feeling of professional isolation and strengthen the competence and autonomy necessary to effect a change in the prestige of the profession (both internally and externally perceived). These platforms should also include allocating time, place and remuneration for such activities as well as redesigning care delivery to clinics with multiple physicians. Developing platforms for interdisciplinary work, particularly with education and mental health staff, could facilitate pediatricians’ ability to adopt more active roles in the community and enhance both their esteem in the eyes of the public and their colleagues. Thus, developing continuing medical education (CME) activities, such as advanced training, courses and webinars should be designed not only to increase knowledge but also to support the three psychological needs pointed out by Self Determination theory (autonomy, competence and relatedness). Such interventions would serve to create the infrastructure to realize the bio-psycho-medical model necessary to the practice of meaningful pediatrics.

## Limitations of research and directions for future research

This study brings a unique perspective on the day-to-day experience of pediatricians working in the community, however, it has some limitations. First, the study is based on a small sample in the survey, and only sparse demographic information was collected. Future research is needed to expand the scope of the study, with the development of an appropriately powered larger scale quantitative survey to ascertain the extent to which these attitudes are prevalent among community pediatricians. The survey could also examine correlations, for example between perceived isolation and community involvement. In addition, the current study focused on pediatricians with experience in community work. Future research should also obtain the views of younger pediatricians that are moving from the hospital to a community setting and the formulation of their professional identity. Furthermore, the theoretical models of career development and professional identity, as well as current research, tend to be apolitical and ahistorical, and do not take into account the role of social power structures. Future studies are needed to examine the links between the development of the professional identity of pediatricians in the community and gender, sector and work in the periphery compared to the center of the country, as well as social and historical context of changes in pediatrics. The change in the professional identity of pediatricians in the community goes hand in hand with historic changes in the field of pediatrics in Israel and around the world.

## Conclusions and implications for policy

Risks to the development and health of children are embedded in the environmental, social and physical conditions in which children live. Promoting and protecting children's wellbeing and health must go beyond the clinic and the treatment of physical illness. The current study examined the way pediatricians in the community perceive their profession, and highlights the variance in perceptions regarding their role and the level of community involvement, as well as the challenges that come with it. Our research findings, despite the scale of the study, reinforce the need for policy change in the field of community pediatrics. As earlier work by Latzer et al. [7] highlighted, developing and applying a community residency that includes the “new morbidities” and relevant skills for working with parents, as well as professional community mentorship, is crucial [7, 8, 27, 28]. However, as highlighted in the current study, transformation is also required within community services. Building a supportive professional community for pediatricians who work in the community is imperative, and should include pediatric coordinators, mentoring by senior pediatricians, regular staff meetings, promoting interprofessional work and CME. The barriers to implementing such changes are manifold, demanding extensive training, increased time for seeing patients with more complex difficulties and resources, along with recognition of need by policy makers. Encouragingly, similar changes have been achieved in relation to family medicine specialization in Israel in recent decades, with creation of a unique residency program, mentoring for young doctors, re-positioning of the profession among doctors and the public, and changes in work practice [29]. Changes of this nature will help in rebranding community pediatrics, should contribute to attracting young physicians to the profession, and ensure better health services for children and families in Israel. Such radical change can only be achieved through partnership between the HMOs, the Ministry of Health, the Scientific Council (Israel Medical Association, professional organizations) and NGOs. In this way the individual-level solutions suggested in this study can be turned into system-level and policy-changing solutions.

## Data Availability

Data are available from the authors upon reasonable request.

## Abbreviations

CME: Continuing medical education
SCCT: Social and Cognitive Career Theory
SDT: Self-determination theory

## Footnotes

1 In Israel many doctors who work primarily in the hospital now supplement their income with work in the community as well.

2 Goshen’s email list includes 1733 pediatricians who work in the community.

3 The Telegram group included about 250 pediatricians at that time, most of them are also on the Goshen email list.
